# Transgenic Tobacco Plants Overexpressing a Grass *PpEXP1* Gene Exhibit Enhanced Tolerance to Heat Stress

**DOI:** 10.1371/journal.pone.0100792

**Published:** 2014-07-08

**Authors:** Qian Xu, Xiao Xu, Yang Shi, Jichen Xu, Bingru Huang

**Affiliations:** 1 National Engineering Laboratory for Tree Breeding, Beijing Forestry University, Beijing, China; 2 Dep. of Plant Biology and Pathology, Rutgers, the State Univ. of New Jersey, New Brunswick, New Jersey, United States of America; TGen, United States of America

## Abstract

Heat stress is a detrimental abiotic stress limiting the growth of many plant species and is associated with various cellular and physiological damages. Expansins are a family of proteins which are known to play roles in regulating cell wall elongation and expansion, as well as other growth and developmental processes. The *in vitro* roles of expansins regulating plant heat tolerance are not well understood. The objectives of this study were to isolate and clone an expansin gene in a perennial grass species (*Poa pratensis*) and to determine whether over-expression of expansin may improve plant heat tolerance. Tobacco (*Nicotiana tabacum*) was used as the model plant for gene transformation and an expansin gene *PpEXP1* from *Poa pratensis* was cloned. Sequence analysis showed *PpEXP1* belonged to α-expansins and was closely related to two expansin genes in other perennial grass species (*Festuca pratensis* and *Agrostis stolonifera*) as well as *Triticum aestivum*, *Oryza sativa*, and *Brachypodium distachyon*. Transgenic tobacco plants over-expressing *PpEXP1* were generated through *Agrobacterium*-mediated transformation. Under heat stress (42°C) in growth chambers, transgenic tobacco plants over-expressing the *PpEXP1* gene exhibited a less structural damage to cells, lower electrolyte leakage, lower levels of membrane lipid peroxidation, and lower content of hydrogen peroxide, as well as higher chlorophyll content, net photosynthetic rate, relative water content, activity of antioxidant enzyme, and seed germination rates, compared to the wild-type plants. These results demonstrated the positive roles of *PpEXP1* in enhancing plant tolerance to heat stress and the possibility of using expansins for genetic modification of cool-season perennial grasses in the development of heat-tolerant germplasm and cultivars.

## Introduction

Plant cell walls provide structural support, control the shape and size of the cell, and protect cells from external biotic and abiotic stresses, and expansin proteins have been shown to control cell wall extensibility and growth [1]. Expansins are small proteins 25–27 kDa in size which bind to glucan-coated cellulose in the cell wall causing a reversible disruption of hydrogen bonding between cellulose microfibrils and the glucan matrix loosening the cell wall [2], [3]. Cho and Kende [4] reported that cell wall extensibility of rice (*Oryza sativa*) coleoptiles was positively correlated with the expression level of an expansin gene, *OsEXP4*. Cho et al. [5] confirmed the role of *OsEXP4* in coleoptile cell wall loosening through sense and antisense expression of *OsEXP4* in rice. Expansins also affect shoot and root elongation [6]–[10] and leaf morphogenesis [11]–[13]. Over-expressing an expansin gene, *AtEXP10*, resulted in increased petiole growth in transgenic *Arabidopsis thaliana* whereas antisense suppressed petiole growth [11].

Expansins may play a role in regulating plant tolerance to abiotic stresses [5], [14]–[17]. At low water potential, several expansin genes were up-regulated in the root-elongation zone of maize (*Zea mays*), which was correlated to root cell elongation and turgor pressure [6], [16], [18]. In rice coleoptiles, *OsEXP2* and *OsEXP4* mRNA induction increased due to flooding stress and northern hybridization results showed that these two expansin genes were correlated with coleoptile elongation in response to oxygen concentration [15]. Transgenic experiments further proved that *OsEXP4* was involved in seedling growth by mediating cell wall loosening [5]. Li et al. [17], [19] identified a drought-related expansin gene *TaEXPB23* and confirmed the functions through over-expression in tobacco (*Nicotiana tabacum*) plants using constitutive and inducible promoters. Limited information is available on expansin regulation of plant tolerance to heat stress. Several studies reported that plant responses to heat stress may involve changes in the expression level of expansin genes. Yang et al. [20] identified an expansin gene homologous to *AtEXP13* which was induced in Chinese cabbage (*Brassica rapa*) seedlings exposed to 37°C for 3 h. Xu et al. [14] identified an expansin gene (*AsEXP1*) from a grass species, which was up-regulated during heat stress in heat-tolerant thermal *Agrostis scabra* but little or no expression in heat-sensitive *A. stolonifera*. Further molecular and physiological analyses revealed that the expression level of *AsEXP1* was positively correlated to whole-plant heat tolerance evaluated as leaf photochemical efficiency and cell-membrane stability in different genotypes of *A. scabra* and *A. stolonifera*. Overexpressing *TaEXPB23* from wheat in tobacco did not result in improvement in heat tolerance [21]. As stated above, expansins are a family of proteins, which are encoded by multiple genes. The differential effects on heat tolerance of different expansins may be due to differences in specific gene functions. The functions of specific expansin genes in conferring plant tolerance to heat stress require further investigation.

One method to determine the role of candidate genes is to manipulate gene expression through transgenic transformation. Transgenic approaches have been successfully used for determining roles of other genes previously identified to be associated with improved heat tolerance in various plant species, such as heat shock proteins, heat shock transcription factors, and antioxidant enzymes [22]–[27]. To our knowledge, the *in vivo* roles of expansins regulating plant tolerance to heat stress have not been well documented. Therefore, the objectives of this study were to isolate and clone an expansin gene in a perennial grass species, Kentucky bluegrass (*Poa pratensis*) and to determine whether over-expression of expansin may improve plant tolerance to heat stress. Tobacco (*Nicotiana tabacum*) was chosen for transformation since previous research has shown it is a simple yet robust method and has been successfully utilized in confirming gene function in various studies. Plant tolerance to heat stress was evaluated through plant growth vigor, as well as cellular and physiological responses of tobacco during heat stress.

## Materials and Methods

### Cloning of a *PpEXP1* gene from *Poa pratensis*

Seeds of Kentucky bluegrass (*Poa pratensis* cv. ‘Diva’) were sown in plastic pots (25 cm deep and 15 cm in diameter) filled with sandy loamy soil. Seedlings were established for 35 d at 25°C and then exposed to heat stress at 40°C in a growth chamber (HP1500 GS-B; Wuhan Ruihua Instrument & Equipment, Wuhan, China). The other growth chamber conditions were 70% relative humidity, 16 h/8 h light/dark cycle, and photosynthetically active radiation (PAR) of 510 mmol m^−2^ s^−1^ during seedling establishment and subsequent heat stress. After 24 h heat treatment, leaves were collected for RNA extraction. Reverse transcription was performed using an oligo (dT)_15_ primer and Moloney murine leukemia virus reverse transcriptase (Promega) at 42°C for 1 h. A primer set was designed for the upstream and downstream sites of the *AsEXP1* gene in *A. stolonifera* (PL7: 5′-CACATTGCTTCTCCCGCTTTTGT-3′, UTR4: 5′-TCGGAGTAGCCGAAGGCCTC-3′), and was used for the homologous gene cloning (*PpEXP1*). The PCR conditions were 94°C for 3 min followed by 32 cycles of 94°C for 1 min, 55°C for 1 min, and 72°C for 1 min. The amplified fragment was cloned into vector pEASY–Blunt (TransGen Biotech). After sequencing, the putative amino acid sequence of the gene *PpEXP1* was deduced using the DNAMAN software (Lynnon Corp. Quebec, Canada). The sequence alignment and phylogenetic tree construction among *PpEXP1* and its homologous genes were analyzed using MEGA 5 [28].

### Construction of the gene over-expression vectors

**Figure 1:**
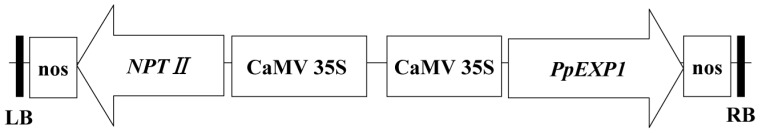
Schematic diagram of the *PpEXP1* over-expression chimeric gene construct, p35S-*NPTII*/35S-*PpEXP1*. *PpEXP1* is under the control of the CaMV 35S promoter and the kanamycin resistance gene *NPTII* is the selection marker.

The full coding sequence (including stop codon) was amplified from the recombinant plasmids with the specific primers for the over-expression vector (PpEXP1: 5′- CCCAAGCTTCAATGGCCTCCTCCAATGC-3′, with added Hind restriction site underlined; PpEXP2: 5′-CCGGAATTCCTAGAACTGGCCTCCTTCG-3′, with added EcoRI restriction site underlined). The amplified fragments and a binary vector pEZR-(K)-LC [29] were both digested with Hind III and EcoRI enzymes and then ligated. The positive recombinant constructs p35S-nptII/35S-PpEXP1 (Fig. 1) were verified using PCR with primers PpEXP1 and PpEXP2 and restriction enzyme digestion with HindIII and EcoRI and constructs were introduced into *Agrobacterium tumefaciens* strain LBA4404 by electroporation transformation.

### Transformation of *PpEXP1* into tobacco plants

Recombinant plasmids were transformed into tobacco plants by *Agrobacterium*-mediated leaf disc infiltration [30]. *Agrobacterium* (LBA4404) carrying the recombinant constructs was cultured in LB medium plus 50 mg L^−1^ kanamycin plates. The plates were incubated for 2–3 days at 28°C. The *Agrobacterium* was then cultured in 30 mL YEP media with appropriate antibiotics at 28°C overnight. The *Agrobacterium* was separated from the YEP medium by centrifuging and the pellets were re-suspended in MS media (OD600 = 0.6∼1.0). Leaf disks (0.5 cm in diameter) of 4–5 week old tobacco plants were incubated in the *Agrobacterium* suspension solution for 10 min. Leaf disks were then blotted dry with sterile paper towels, and cultured in MS solid media in the dark at 28°C to generate transgenic plants.

Shoot regeneration was induced on Tobacco Selection Medium (30 g L^−1^ sugar, 4 g L^−1^ phytagel, 3 mg L^−1^ 6-BA, 0.2 mg L^−1^ NAA, 100 mg L^−1^ kanamycin, 250 mg L^−1^ sodium cefotaxime in Murashige and Skoog medium) [31]. After five weeks, the regenerated shoots were transferred to a root-inducing medium (Murashige and Skoog medium with 30 g L^−1^ sugar, 4 g L^−1^ phytagel, 100 mg L^−1^ kanamycin, 250 mg L^−1^ sodium cefotaxime). The positive transformants were verified using PCR with the primer pair PpEXP1/PpEXP2.

### Germination test of the transgenic and wild type tobacco seeds with the heat treatment

Seeds of the T0 transgenic line and wild type (WT) plants were wrapped in wet paper towels in petri dishes and incubated at 30, 40, or 50°C for 2 h in an incubator. Seeds were then placed on MS medium and kept at 28°C for germination. The percentage of germinated seeds from the total number of seeds for the transgenic line or the wild type was determined.

### Physiological analysis of transgenic and the wild type tobacco plants in heat stress

Positive transgenic tobacco seedlings were planted in pots filled with vermiculite. Plants were irrigated daily and fertilized weekly with half-strength Hoagland's nutrient solution [32]. Two-week old seedlings of the transgenic and wild-type (WT) plants were exposed to heat stress (42°C) or the optimal growth temperature (25°C) for 6 d with other environmental conditions the same as previously described. Leaves were destructively sampled for the physiological tests following 6 d of treatment. Several physiological parameters were measured to evaluate heat tolerance in the wild type and transgenic lines, including leaf cell membrane stability, relative water content, chlorophyll content, net photosynthetic rate, membrane lipid peroxidation, content of hydrogen peroxide, and antioxidant activity of superoxide dismutase.

Cellular membrane stability was evaluated by measuring relative electrolyte leakage (EL) of leaves using the methods of Blum and Ebercon [33]. For EL measurement, leaf disks (1.2 cm in diameter) of 0.2 g fresh weight were taken from three leaves per plant and incubated in 40 mL deionized water for 12 h on a conical shaker. The conductance of the solution was measured as the initial level of conductance (C_i_) using a conductance meter (Yellow Springs Instrument Co., Ohio, USA). Leaf tissues were then killed in an autoclave at 121°C for 15 min. After 12 h incubation on a conical shaker, the conductance of the sample was measured again as C_max_. Leaf EL was calculated as: EL (%) = C_i_/C_max_×100.

Leaf relative water content was calculated using fresh weight (FW), turgid weight (TW), and dry weight (DW) of leaves according to the equation: (FW-DW)/(TW-DW)×100 [34]. Fresh weight was measured immediately after leaves were cut off the plant. After FW determination, leaves were soaked in distilled water for 12 h at 4°C until leaves became fully turgid, and then blotted dry to determine TW. Leaf DW was measured after leaves were dried in an oven at 87°C for 72 h.

For leaf chlorophyll content, chlorophyll was extracted from leaf tissue of 0.1 g fresh weight in dimethyl sulfoxide and kept in the dark for 2 d. The absorbance of the solution was determined at 645 and 663 nm using a spectrophotometer. Leaf chlorophyll content was calculated using the equation described in [35].

Leaf net photosynthetic rate was measured with an infrared gas analyzer (LI-6400, LICOR Inc., Lincoln, NB). A single leaf was enclosed in the leaf chamber (2×3 cm) and exposed to light (PAR of 650 µmol photon m^−2^ s^−1^) with a built-in red and blue light source of the LI-6400 and carbon dioxide concentration controlled at 380 µmol m^−1^). Leaf net photosynthetic rate was measured at 10:00 AM.

Membrane lipid peroxidation was determined by measuring malondialdehyde (MDA) content following the procedure described in [36]. Briefly, fresh leaves (0.2 g) were ground in 10% trichloroacetic acid containing 0.5% thiobarbituric acid. The mixture was heated in a water bath at 95°C for 30 min, quickly cooled on ice, and then centrifuged at 14,000 g for 20 min. The absorbance of the supernatant was measured at 532 and 600 nm using a spectrophotometer. The concentration of MDA was calculated using an extinction coefficient of 155 mM^−1^ cm^−1^ [37].

The content of hydrogen peroxide was determined following the method described in [38] with modification. Leaf samples were extracted in acetone. The supernatants (1 mL) were added in a reaction solution containing 0.1 mL 20% titanium reagent (20% w/v TiCl_4_ in 12.1 mL HCl) and 0.2 mL 17 M ammonia. The solution with the extractants was centrifuged at 3000 g at 4°C for 10 min and the pallets were dissolved in 3 mL 1 M sulfuric acid. The absorbance of the solutes was measured at 410 nm with a spectrophotometer.

The activity of an antioxidant enzyme, superoxide dismutase (SOD), was measured using the method previously described in [39] with modification. Briefly, 0.2 g of fresh leaf tissue was ground to a fine powder using a mortar and pestle and extracted in 4 mL of extraction buffer at pH 7.8 containing 50 mM potassium phosphate, 1 mM ethylenediaminetetraacetic acid, 1% polyvinylpyrrolidone, 1 mM dithiothreitol, and 1 mM phenylmethylsulfonyl. The extractants were centrifuged at 15,000 g for 30 min at 4°C, and supernatant was collected for enzyme assay. The SOD (EC 1.15.1.1) activity was measured by recording changes in the rate of nitro blue tetrazolium chloride reduction in absorbance at 560 nm.

### Cellular structural changes of transgenic and wide-type tobacco plants under heat stress

The 5 mm leaf segments were fixed by immersion in newly- prepared neutral sodium phosphate (0.1 M) with 5% paraformaldehyde at 4°C overnight. After washing in phosphate buffer, leaf samples were dehydrated in a series of ethanol solutions at concentration of 30%, 50%, 70%, 85%, 95%, and 100%. The dehydrated tissues were embedded and polymerized in Epon812 resin at 70°C. Leaf samples were then cut horizontally into 2 µm-thin sections and stained by toluidine blue. The images of leaf cross sections were recorded by a camera fitted to a microscope (Nikon, Tokyo, Japan).

### Statistical analysis

Data for effects of temperature and transformation on physiology and germination rate were determined using the analysis of variance (ANOVA) with a general linear procedure. Significance between treatments was determined using the least significance test (LSD) at p = 0.05 with a statistical program (SAS Version 9.0, Cary, NC).

## Results

### Sequence characteristics and expression of *PpEXP1*

**Figure 2:**
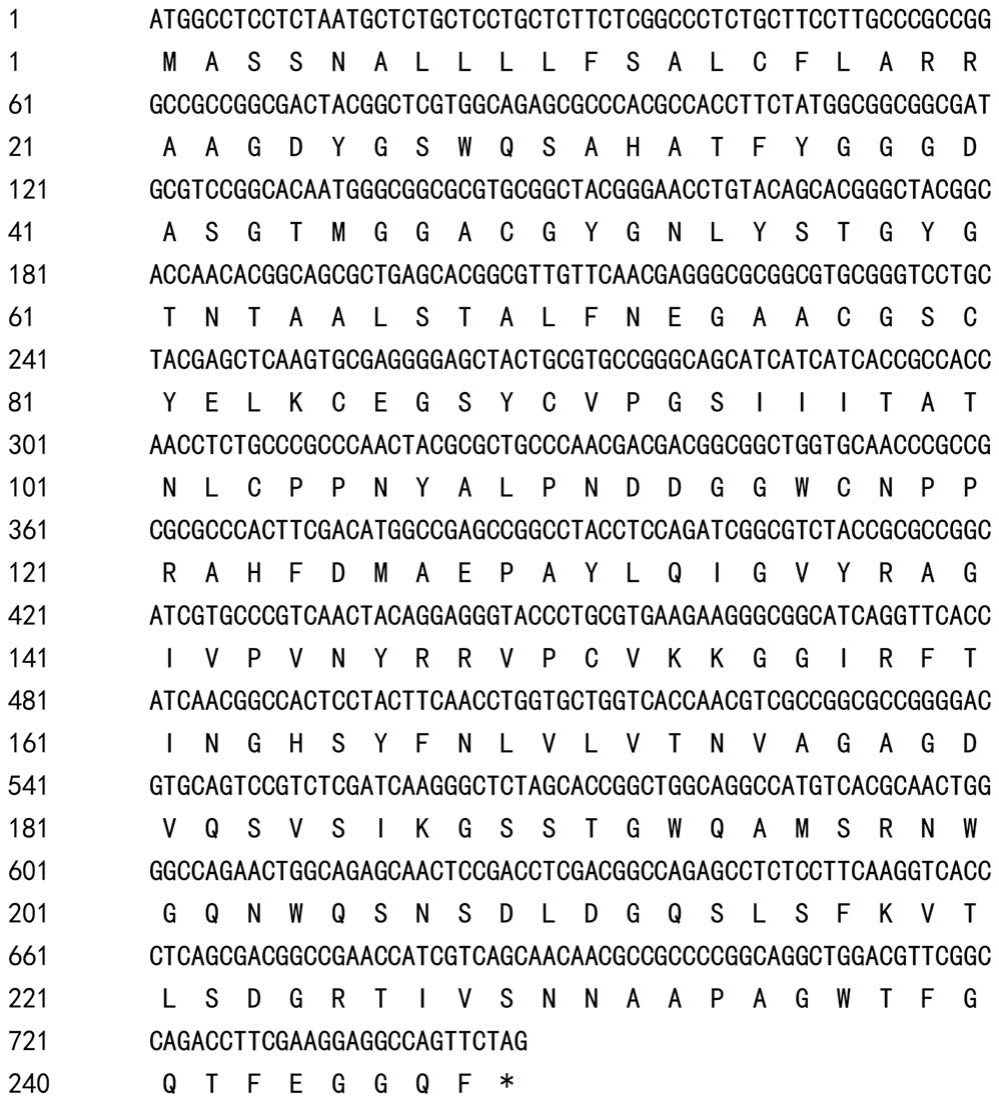
The full sequence of *PpEXP1* gene from *Poa pratensis* with the deduced amino acids.

**Figure 3:**
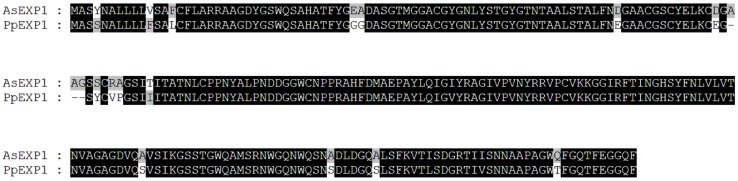
The protein sequence alignment of *PpEXP1* from *Poa pratensis* and *AsEXP1* from *Agrostis stolonifera*.

**Figure 4:**
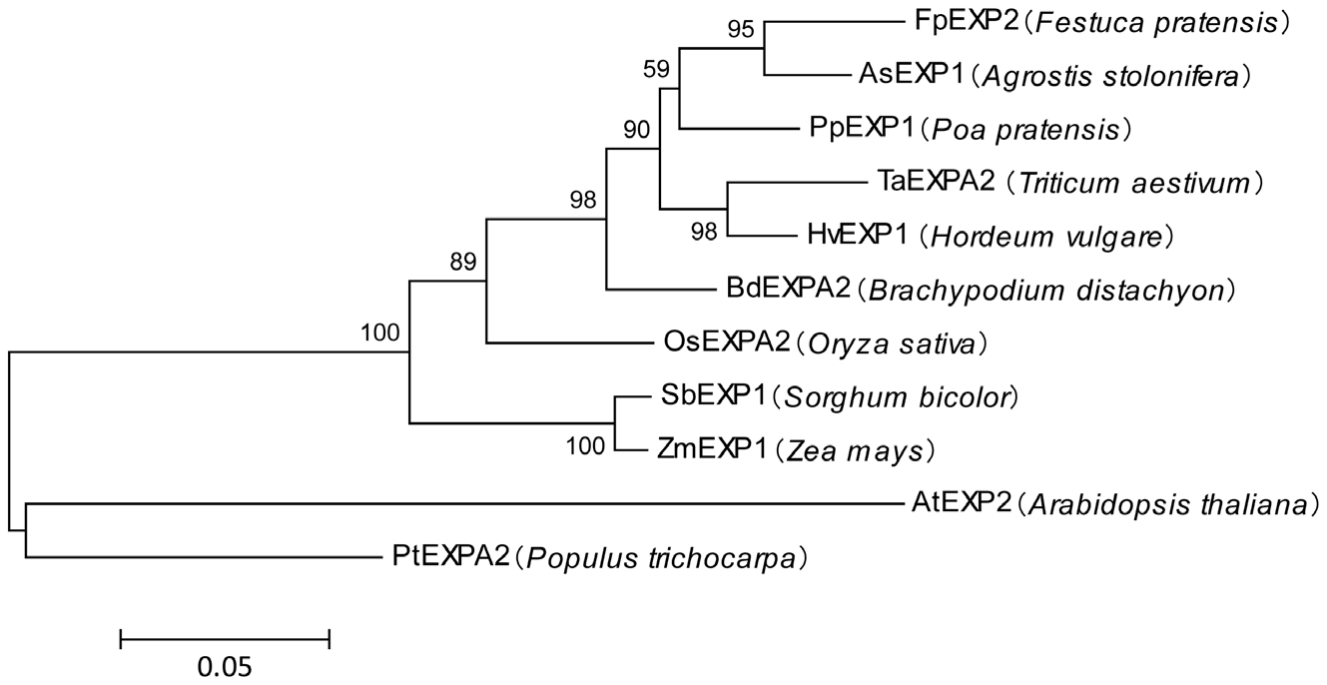
A phylogenetic tree constructed with MEGA5 based on the amino acids of the *PpEXP1* homologous genes from different plant species. Accession numbers of these proteins listed below: FpEXP2 (CAC06433), TaEXPA2 (AAT94292), HvEXP1 (BAK05504), BdEXPA2 (XP_003564501), OsEXPA2 (NP_001044656), SbEXP1 (XP_002456550), ZmEXP1 (NP_001105040), AtEXP2 (XP_002871148), PtEXPA2 (XP_002319409). Sequence of AsEXP1 was from Zhou et al. [47].

Using the primers designed based on the sequence of *AsEXP1*, the homologous gene fragment was amplified and cloned from the transcript of *Poa pratensis* plants exposed to heat stress and named as *PpEXP1*. Sequencing results showed the gene's open reading frame contained 744 nucleotides (bp) encoding 247 amino acids (Fig. 2). The amino acid alignment shows an identity of up to 92% between *PpEXP1* and *AsEXP1* from *A. stolonifera* (Fig. 3) with a 3 amino acid deduction in *PpEXP1*. Searches in Genebank for homologous genes revealed several matches including 8 monocots and 2 eudicots. A phylogenetic tree was constructed based on their amino acid sequences and shown in Fig. 4. The monocot and eudicot plants were basically distributed into two branches. The C_3_ plants (*Triticum aestivum*, *Oryza sativa*, *Festuca pratensis*, *Poa pratensis*, *Agrostis stolonifera*, and *Brachypodium distachyon*) and C_4_ plants (*Zea mays* and *Sorghum bicolor*) of the 9 monocots species were classified into two sub-clades. The *PpEXP1* gene was more closely grouped with two other perennial grass species (*F. pratensis* and *A. stolonifera*), *Triticum aestivum*, *Hordeum vulgare*, and *Brachypodium distachyon* based on the gene sequences. The topology structure fits well to the phylogenetic tree constructed by whole chlorophyll genome sequence and molecular markers [40], [41].

**Figure 5:**
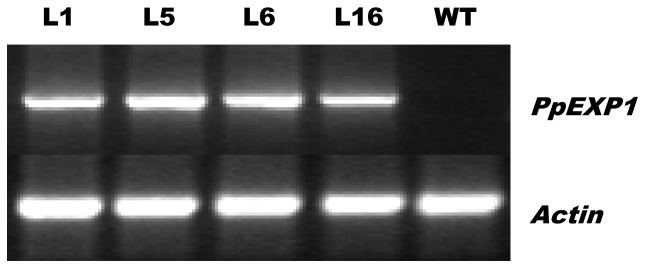
*PpEXP1* gene expression in transgenic (L1, L5, L6, L16) and wild type (WT) tobacco by RT-PCR.

Transgenic lines of tobacco obtained using agrobacterium-mediated transformation were tested at the RNA level using the specific primers of PpEXP1/PpEXP2. *PpEXP1* expression was confirmed in all transgenic lines using RT-PCR. The expression of *PpEXP1* in four transgenic tobacco lines (L1, L5, L6, and L16) is shown in Fig. 5.

### Seed germination rate of transgenic and WT plants as affected by heat stress

The T1 seeds for the WT and four transgenic lines (L1, L5, L6, and L16) were harvested and used for the germination test. Seed germination rate decreased with increasing temperature from 30 to 40 and 50°C in the WT (Table 1). Seed germination rate did not change in any of the four transgenic lines as temperature increased from 30 to 40°C, while the decline was observed at 50°C in L1 and L5. Increasing temperatures did not have significant effects on the seed germination rates of L6 and L16. Seed germination rates of all four transgenic lines were significantly higher than that of the WT at 40 and 50°C.

**Table 1:** Germination rate of seeds from transgenic lines and wide-type tobacco plants treated at different temperatures.

	Germination rate (%)				
Treatment	L1	L5	L6	L16	WT
30°C	92.3±1	94.2±1	93.6±0.6	92.3±1	91.7±0.6
40°C	91.0±0.6	92.3±1	92.9±0.6	91.0±0.6	76.3*±0.6
50°C	87.2±0.6	89.1±0.6	90.4±1	88.5±1	60.9*±0.6

All data are mean ± SD of each treatment. Three replicates were performed for each treatment.

“*” indicates significant difference of the WT data from all transgenic lines at 40 or 50°C based on least significance difference test at p  = 0.05. Data at 30°C without “*” indicate no significant differences among the WT and transgenic lines.

### Plant vigor and cellular structural changes of transgenic and WT plants in response to heat stress

**Figure 6:**
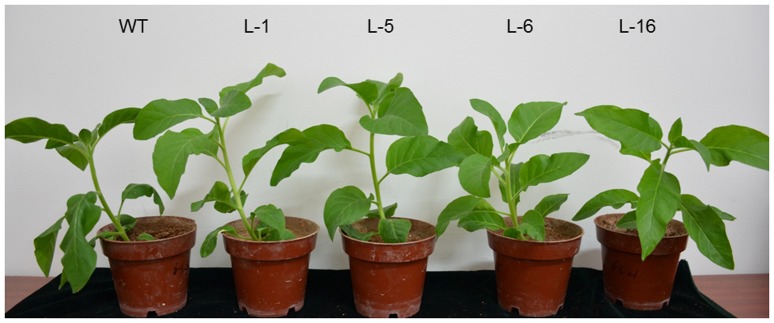
The transgenic (right) and wild-type (left) tobacco plants were under heat treatment of 42°C for 6 d, which the leaf wilting was rating as 8 and 4, respectively.

Plants of four transgenic lines (L1, L5, L6, and L16) and the WT were exposed to heat stress to evaluate growth responses. Leaf wilting was observed in the WT plants at 3 d of heat stress, but was not seen in any of the transgenic lines. After 6 d of heat stress, the WT plants exhibited severe leaf wilting, chlorosis of mature leaves, and stunted growth, but the transgenic plants did not exhibit signs of heat damages as seen in the wild type (Fig. 6). Plant vigor was rated based on the level of leaf wilting, leaf color, and canopy height on the scale of 1–9, with 9 being undamaged and 1 being a dead plant. The rating was 4±0.5 (n = 10) for the WT plants and averaged at 8±0.5 (n = 10) for the four transgenic lines.

**Figure 7:**
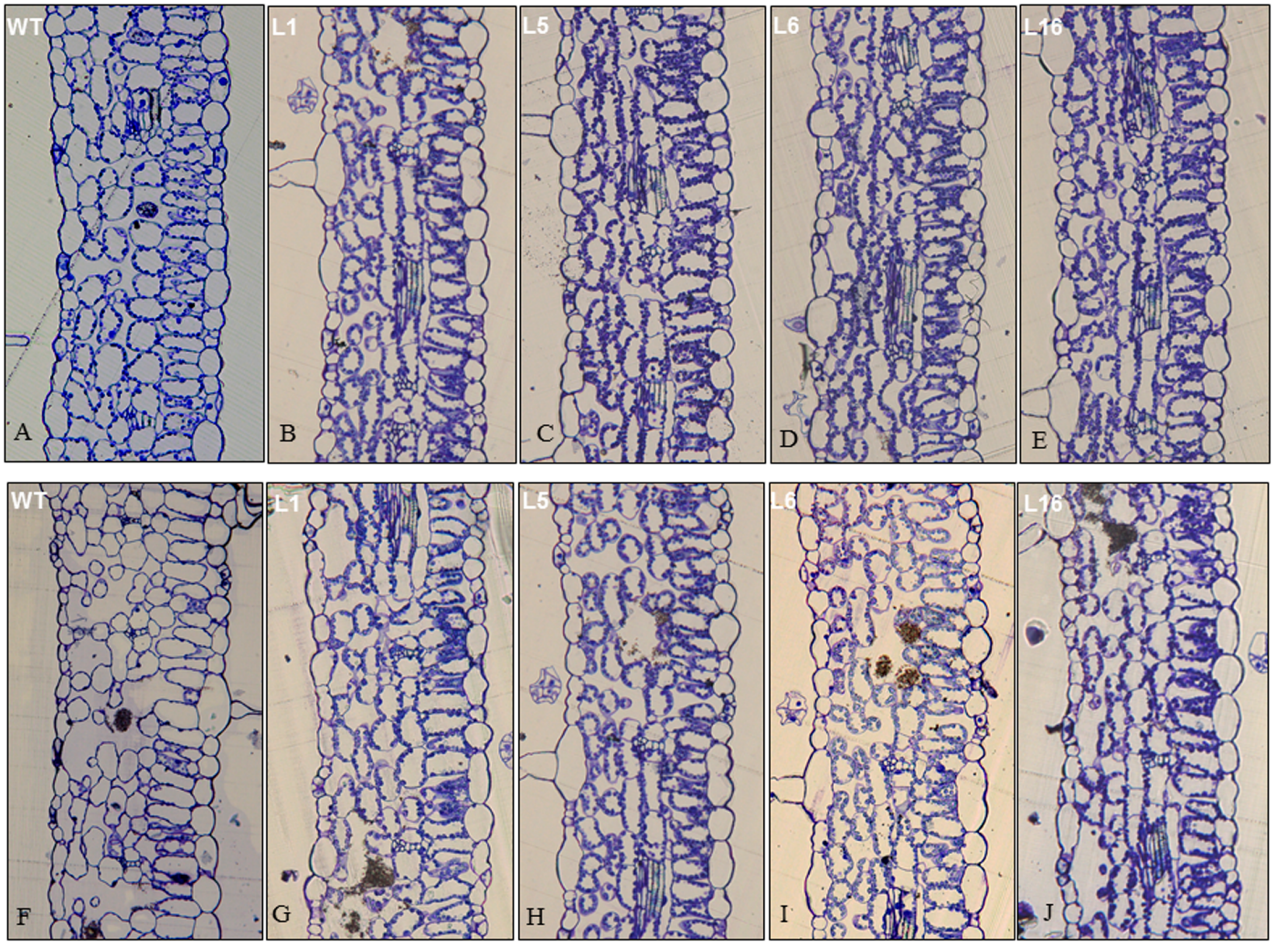
Transverse sections of leaf tissues from WT and transgenic tobacco plants and imaged captured on a camera fitted to a microscope at 20 X. A, WT 25°C; B, 35S::*PpEXP1* 25°C; C, WT 42°C; D, 35S::*PpEXP1* 42°C.

Cellular damages in transverse sections of leaf tissues from the WT and transgenic plants exposed to the optimal growth temperature and heat stress were examined by microscopy (Fig. 7). Under the optimal growth temperature (25°C), mesophyll cells were intact while palisade and spongy mesophyll cells were arranged orderly with a clearly-defined cellular structure in leaves of WT (Fig. 7A) and four transgnic lines (Fig. 7B–E). After heat treatment at 42°C for 6 d, the WT plants displayed some cellular disruption and disorganization of the spongy mesophyll cells with large intercellular spaces, which could be the result of cell death (Fig. 7F). Heat-stressed transgenic plants still maintained well-defined mesophyll cells and transversal structures of the leaf were similar between leaves exposed to the optimal growth temperature and heat stress (Fig. 7G–J). Plastids were present as granules along the mesophyll cell walls in both the WT and transgenic plants grown at the optimal growth temperature (Fig. 7A and 7B–E). Under heat stress, majority of plastids disappeared in the WT leaves (Fig. 7F) but remained intact in high density in the transgenic leaves (Fig. 7G–J).

### Physiological changes of the transgenic and WT plants in response to heat stress

**Figure 8:**
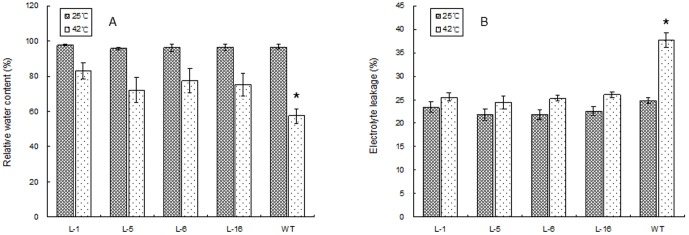
Leaf relative water content (A) and electrolyte leakage (B) in WT and four lines of transgenic tobacco under normal temperature and at 6 d of heat treatment. WT represents the wild type tobacco. L1, L5, L6, and L16 are different transgenic tobacco lines. The vertical bar over each column represents standard error of the mean for three replicates in each treatment. The “*” over the column of the WT indicates significant difference of the WT from all transgenic lines under heat stress based on least significance difference test at p = 0.05. Columns without “*” indicate no differences among the WT and transgenic lines.

**Figure 9:**
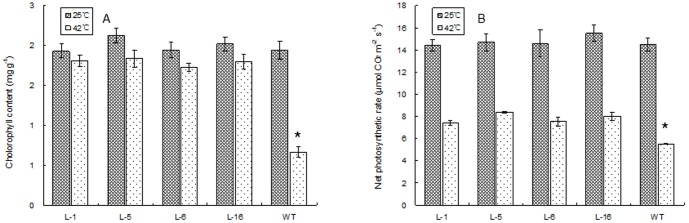
Leaf chlorophyll content (A) and net photosynthetic rate (B) in WT and four lines of transgenic tobacco under normal temperature and at 6 d of heat treatment. WT represents the wild type tobacco. L1, L5, L6, and L16 are different transgenic tobacco lines. The vertical bar over each column represents standard error of the mean for three replicates in each treatment. The “*” over the column of the WT indicates significant difference of the WT from all transgenic lines under heat stress based on least significance difference test at p = 0.05. Columns without “*” indicate no differences among the WT and transgenic lines.

**Figure 10:**
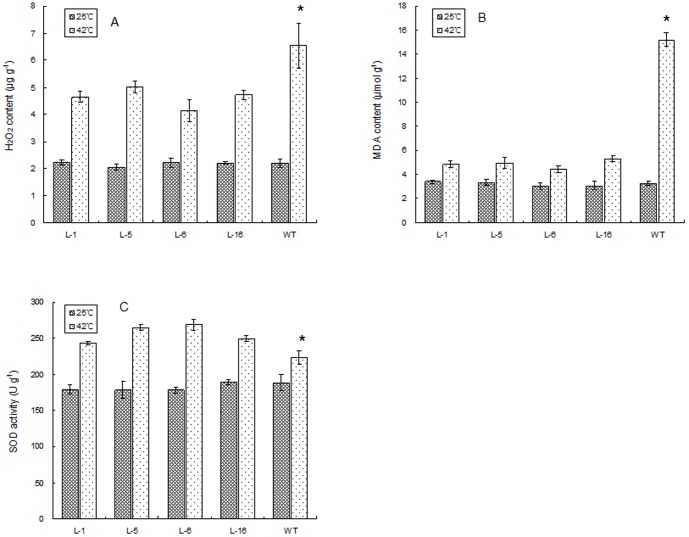
Hydrogen peroxide (H_2_O_2_) content (A), and malondialdehyde (MDA) content (B), and activity of superoxide dismutase (SOD) (C) in WT and four lines of transgenic tobacco under normal temperature and at 6 d of heat treatment. WT represents the wild type tobacco. L1, L5, L6, and L16 are different transgenic tobacco lines. The vertical bar over each column represents standard error of the mean for three replicates in each treatment. The “*” over the column of the WT indicates significant difference of the WT from all transgenic lines under heat stress based on least significance difference test at p = 0.05. Columns without “*” indicate no differences among the WT and transgenic lines.

Under the optimal temperature conditions, no significant differences in any of the physiological parameters (Fig. 8, 9, 10) were detected between the four transgenic lines and the WT plants. At 6 d of heat stress, relative water content (Fig. 8A), chlorophyll content (Fig. 9A), and net photosynthetic rate (Fig. 9B) declined in all plants, to a lesser extent for all three parameters in the four transgenic lines than the WT plants. Leaf EL (Fig. 8B),H_2_O_2_ content (Fig. 10A), and MDA content (Fig. 10B), as well as SOD activity (Fig. 10 C)increased significantly at 6 d of heat stress in all plants, and the magnitude of increase for EL, H_2_O_2_, and MDA was less pronounced in the four transgenic lines than in the WT plants. Four transgenic lines maintained significantly higher relative water content, chlorophyll content, and net photosynthetic rate, and SOD activity, but had lower EL, H_2_O_2_, and MDA content compared to the WT.

## Discussion

Expansins include two major types, α –expansins and β-expansins, which are encoded by a large gene family [42]. As shown in Fig. 4, the *PpEXP1* gene was grouped closely with two other perennial grass species (*F. pratensis* and *A. stolonifera*), as well as other monocots including *Triticum aestivum*, *Oryza sativa*, and *Brachypodium distachyon*, but was less related to *Arabidopsis thaliana* based on the gene sequence of the current study. The functional conserved amino acids and motifs specific to α-expansin were found in *PpEXP1* proteins, such as cysteines that form the disulfide bridges, HFD motif in the GH45-like domain and the aromatic residues in the binding-domain, suggesting that *PpEXP1* is belong to α-expansin family. The α-expansins are predominantly found in dicots, and also found in monocots [42]. In addition, the amino acid sequence alignment showed a 92% similarity to the reported *AsEXP1*sequences in *A. stolonifera* [14]. These data suggest that *PpEXP1* is a conserved α-expansin gene in grass species.

Over-expression of *PpEXP1* in tobacco enhanced seed germination during heat stress and suppressed heat-induced leaf damages by maintaining higher leaf chlorophyll content, relative water content, net photosynthetic rate, antioxidant enzyme activity, and lower EL, H_2_O_2_ and MDA content (Fig. 8, 9, 10). These results suggested that *PpEXP1* could be involved in heat adaptation by promoting leaf photosynthesis and hydration status, and strengthening antioxidant activities to suppress the accumulation of reactive oxygen species and membrane damages. Less cellular disruption during heat stress was seen during microscopic observations of cells in the transgenic plants (Fig. 7). Cell death associated with cell structural damages, such as rupture of cell membranes and cell walls are typical heat stress symptoms in plant cells [43]. Loss of mesophyll cellular structure and presence of large intercellular spaces were shown in the WT plants under heat stress. However, the mesophyll cells in leaves of *PpEXP1* transgenic plants were maintained with well-defined cellular structure and a high density of plastids. These results indicated that over-expression of *PpEXP1* could facilitate cellular survival during heat stress and possibly be linked to the effects on maintaining cell wall extensibility. Previous research has shown that heat treatments inactivate expansins and increase cell wall rigidity [44]. The cellular observations were in line with the physiological data, supporting the positive effects of *PpEXP1* over-expression on heat tolerance. Leaf chlorophyll content, net photosynthetic rate, and water status have been shown to be positively correlated to plant heat tolerance while EL and oxidative damages due to increased H_2_O_2_ and MDA production are negatively correlated to plant heat tolerance [45], [46]. The expression level of *AsEXP1* was previously found to be positively correlated to heat tolerance in two grass species contrasting in heat tolerance. Higher expression of expansin in the heat-tolerant thermal *A. scabra* relative to heat-sensitive *A. stolonifera* was associated with significantly higher leaf chlorophyll content and lower electrolyte leakage under heat stress in the former compared to the latter species [47]. To our knowledge, this is the first study that demonstrated the positive effects of over-expressing α-expansin, as *PpEXP1*, on improving heat tolerance. Our results suggest that expansins play important roles in protecting membranes and reducing cellular and physiological damages from heat stress, thereby improving plant tolerance to heat stress.

The underlying mechanisms of expansin genes regulating plant tolerance to heat stress are not well understood. Cell wall extensibility regulated by expansin can be affected by external stresses, including temperature [44]. Despite the knowledge of the positive association of expansin expression and plant heat tolerance, the specific effects of high temperatures on cell-wall properties as affected through the regulation of expansins is not clear. Heat treatment inactivates expansins in the cell wall, leading to the reduction in cell wall extensibility and the cell wall extensibility can be recovered by increasing expansin proteins in the heat-treated cell walls [44]. The correlation of expansin and cell wall extensibility has been confirmed in various plant species, including tomato leaves (*Solanum lycopersicum*) [48], oat (*Avena sativa*) coleoptiles [49], rice (*Oryza sativa*) internodes [50], and soybean (*Glycine max*) roots [7]. The abnormal cell growth of *Arabidopsis* plants exposed to high temperature has been associated with the loss of cellulose crystalline and cell wall extensibility [51]. Based on the functions of expansins in the cell wall it could be assumed that *PpEXP1* may be involved in protecting cellular damages from heat stress by loosening cell walls, and maintaining cellular turgor and cell integrity under heat stress. This notion deserves further investigation using the transgenic plants over-expressing *PpEXP1*.

In summary, *PpEXP1* cloned from Kentucky bluegrass was classified as an α-expansin and closely related to expansin genes in other grass species. Over-expressing *PpEXP1* in tobacco mitigated cellular damages and growth inhibition due to heat stress and improved heat tolerance of tobacco as manifested by greater seed germination rate, chlorophyll content, and cell membrane stability, as well as lower level of membrane lipid peroxidation. However the cellular and molecular mechanisms of PpEXP1 regulating plant heat tolerance requires further investigation. The effectiveness of *PpEXP1* in grass heat tolerance will be investigated in future research through over-expressing the gene in cool-season perennial grasses that are sensitive to heat stress. *PpEXP1* is a potential candidate gene that could be used to generate heat-tolerant cool-season perennial grass germplasm and cultivars, such as used in forage and turf grasses.
